# On Gout

**Published:** 1805-04-01

**Authors:** 


### To the Editors of the Medical and Physical Journal.

GENTLEMEN,

THE melancholy case of gout, detailed by Mr. O'Neal in the last number of your Journal, forces from me some animadversions on the rash practice which was employed in it, and which, I hope, will be a warning to those who favour Dr. Kinglake's mode of treatment, how they persist in cold applications, heedless of the appearance of dangerous symptoms, until it is too late to reclaim their hasty measures. In justice indeed to Dr. Kinglake, I think the practice in this case was injudiciously carried much farther than he himself would have ventured upon; and that, in his remarks on the case, he pays too great a deference to Mr. O'Neal's opinion, in referring to apoplexy, symptoms, which every unbiassed practitioner will pronounce to have been repelled gout. Indeed, Gentlemen, you must pardon my surprize at your delay in publishing Mr. O'Neal's case (which is dated in September last), when cases of fortunate termination are immediately inserted in your Journal; and I shudder to think how many fatal cases may have unfortunately occurred from the indiscriminate application of cold, which an earlier insertion of this case might have prevented. I hope I am not mistaken, when I view in Mr. O'Neal a youthful enthusiast, laudably ardent in the cause of science and humanity, but whom experience hath not yet taught to adopt, with limitation and caution, such opinions and practices as run counter to the observation of many ages. If, with experience as yet necessarily limited from my years, I may be allowed to quote myself, I would mention that I formerly used, without the proper cautions so judiciously laid down by Dr. Currie, the cold treatment in fevers, of the good effects of which, under these restrictions, I am daily more convinced by farther trial; and have now only the consolation of believing, that although the cold was applied in judiciously and with bad effect, the cases were of such a type, and in such patients as would most probably, under any treatment, have terminated fatally. I too am an advocate for Dr. K's practice in a limited sense, and am far from taxing him, as Mr. Edlin (except he had produced some proof of his assertion) has uncandidly done, with giving fictitious cases; but I will say, that I view with distrust some of his narrations, as I would do those of every man who has attached himself strongly to a particular hypothesis; knowing how much a favourite opinion stands before and obscures the correct and dispassionate judgment of even the keenest observer. Many parts of his theory I profess I do not understand, and cannot therefore accede to. To some sentences in his work I can attach no meaning whatever. That cold is indicated, and may be safely applied in young robust subjects under the first paroxysms of gout, when the inflammation of the foot runs high, and where there has never been shown any atony or great derangement of the stomach, or other vital viscus, I must admit, and would give Dr. K. every credit for so far introducing this practice to public attention. I would also give it as my opinion, that thereby the paroxysm being shortened, the patient will perhaps be spared a long confinement, and the parts will be left in a less debilitated state, and with less disposition to a return of the disease, provided the proper general regimen be strictly pursued. But I think such cases are comparatively but few. Without disputing with the doctor about the diversity of the structure of parts, and the gouty inflammation being confined to those of a particular conformation, and letting others argue as they please about the existence and nature of gouty matter, and upon other points in dispute, which perhaps the present state of pathological knowledge, whatever it may afterwards become, will not yet permit us to solve; I would only request his attention to the following questions, and the serious inquiry of every practitioner into these and other phænomena of the disease, before they prevent it from wasting its accumulated influence on parts least essential to life. Are not most attacks of gout in the limbs, especially after the first or second, and when the patient's constitution has previously been impaired by the disease, preceded by more or less affection of the stomach, consisting generally in atony, impaired digestion, and disturbed action of the chylopoetic organs and consequent derangement of the whole system, which symptoms are relieved or disappear on one or more of the joints becoming affected with the gouty inflammation? Does not this inflammation sometimes suddenly disappear, and a train of alarming symptoms immediately ensue; either in the head, and consisting of coma, and a loss of sensation and motion; or, as most frequently happens, in the stomach, indicated by a torpor, or even total suspension of the powers of that organ, which if it continues for any time terminates in death? Is not this fatal retrocession often often the effect of cold and other repellents (I use these terms in a general sense, as convenient, without wishing to involve any particular theory) injudiciously applied soon after the gouty inflammation has appeared, or before it seems disposed to fix itself, and is yet flying from joint to joint? If these alarming symptoms have not proceeded far, and are combated in time, do they not cease on the re-appearance of the inflammation, or swelling externally? Have we not all heard patients themselves, people unbiassed by hypothesis, in an early stage of the paroxysm, and while the pains were yet flying about, declare, that if by chance they exposed an arm or leg for any time to the cold air, they immediately felt an uneasy sensation and *sinking* at the stomach? When indisputable cases of the most unfortunate sequel occur, are even numberless cases terminating favourably to warrant more than a very guarded practice? Was not the disease in the unfortunate case under consideration, successively driven from the extremity to the stomach and head by the means employed? And might not a valuable officer have been saved to his country, by allowing the inflammation to fix in the foot, as it seemed disposed, and by moderately indulging the salutary craving of nature to support the tone of the stomach?

*March* 5, 1805. R. S.

